# Binocular metamorphopsia in patients with branch retinal vein occlusion: a multi-center study

**DOI:** 10.1007/s10792-023-02731-0

**Published:** 2023-05-25

**Authors:** Rie Osaka, Yuki Muraoka, Daisuke Nagasato, Yoshinori Mitamura, Naomi Nishigori, Tomoaki Murakami, Kiyoshi Suzuma, Hitoshi Tabuchi, Akitaka Tsujikawa

**Affiliations:** 1 Department of Ophthalmology, Faculty of Medicine, Kagawa University, Kagawa, Japan; 2 Department of Ophthalmology and Visual Sciences, Kyoto University Graduate School of Medicine, Kyoto, Japan; 3 Department of Ophthalmology, Saneikai Tsukazaki Hospital, Himeji, Japan; 4 Department of Technology and Design Thinking for Medicine, Hiroshima University Graduate School, Hiroshima, Japan; 5 Department of Ophthalmology, Institute of Biomedical Sciences, Tokushima University Graduate School, Tokushima, Japan

**Keywords:** Metamorphopsia, Binocular metamorphopsia, M-CHARTS, Macular edema, Branch retinal vein occlusion

## Abstract

**Purpose:**

The pathology of branch retinal vein occlusion (BRVO), a retinal circulatory disease, is related to monocular metamorphopsia-related vision impairment of the affected eyes, but the association of binocular metamorphopsia in such patients is unclear. This study aimed to examine the frequency of binocular metamorphopsia and its association with the clinical characteristics of patients with BRVO.

**Methods:**

A total of 87 patients who were treated for BRVO-associated macular edema (ME) were included in this study. At baseline and 1 and 3 months after the initiation of anti-vascular endothelial growth factor (VEGF) treatment, we quantified metamorphopsia in the affected eyes and binocular metamorphopsia using the M-CHARTS^®^ diagnostic tool.

**Results:**

At baseline, 53 and 7 patients had metamorphopsia in the affected eyes and binocular metamorphopsia, respectively. Although the visual acuity improved significantly after the initiation of anti-VEGF treatment, the mean M-CHARTS score in the affected eyes did not change from the baseline score. At 3 months, 9 patients showed binocular metamorphopsia; it was significantly associated with metamorphopsia in the affected eyes with a 95% confidence interval of 0.021–0.122 (β = 0.306, *p* = 0.006).

**Conclusion:**

Metamorphopsia in the affected eyes can cause binocular metamorphopsia in patients with BRVO-ME.

**Supplementary Information:**

The online version contains supplementary material available at 10.1007/s10792-023-02731-0.

## Introduction

Branch retinal vein occlusion (BRVO) is the second most common retinal circulatory disease, and causes the retinal edema, hemorrhage, and/or ischemia in the affected area [1, 2]; involvement of the macular area in these pathological indications results in significant vision impairments [3–5]. Anti-vascular endothelial growth factor (VEGF) therapy can be used to treat most macular edemas (MEs) [6–10], and this has led to substantial improvements in the clinical management of decreased visual acuity (VA) [11–13].

Previous studies have shown that approximately 90% of patients with BRVO-ME suffer from monocular metamorphopsia-related vision impairment of the affected eyes [14]. Furthermore, it has also been elucidated that monocular metamorphopsia after BRVO-ME could negatively affect vision-related quality of life [15–17]. In clinical practice, occasional cases of patients with BRVO who complained of visual disability were observed, and this possibly could be related to metamorphopsia of the affected eyes [18, 19].

To better evaluate the association between the vision impairments and visual disability in patients with BRVO, a routine examination only for the affected eye may not be sufficient [20–22] and requires additional assessment (in addition to monocular metamorphopsia) of binocular metamorphopsia. However, the occurrence of binocular metamorphopsia in patients with BRVO is not fully investigated yet. This study aimed to examine the mono- and binocular metamorphopsia and associated clinical factors in patients with unilateral BRVO-ME.

## Methods

This study adhered to the tenets of the Declaration of Helsinki and was approved by the Ethics Committees of the Kagawa University Faculty of Medicine (Kagawa, Japan), Kyoto University Graduate School of Medicine (Kyoto, Japan), Tokushima University Faculty of Medicine (Tokushima, Japan), and Saneikai Tsukazaki Hospital (Hyogo, Japan). Written informed consent was obtained from each patient before any study procedure or examination.

### Patients

A total of 87 patients with unilateral and treatment-naïve BRVO who were examined and treated at Kagawa, Kyoto, Tokushima University, and Tsukazaki Hospital from April 2018 to March 2019 were included in this study. The inclusion criteria were as follows: (1) symptomatic BRVO with retinal hemorrhage and edema involving the macula, (2) foveal thickness (FT) greater than 250 µm at baseline (as measured by optical coherence tomography [OCT]), and (3) symptom duration of less than 3 months prior to the initial examination. BRVO was diagnosed based on fundus examinations, OCT angiography (PLEX Elite 9000; ZEISS, Germany), and/or fluorescein angiography (FA) findings. We excluded eyes with central retinal vein occlusion (CRVO) or hemi-CRVO and co-morbid ocular diseases that could affect the macula (e.g., epiretinal membrane, macular hole, diabetic retinopathy, retinal arterial macroaneurysm, glaucoma, central serous chorioretinopathy, age-related macular degeneration, and retinitis pigmentosa). We also excluded eyes with dense cataracts that could compromise OCT or OCT angiography image qualities and previously received any ocular interventions other than the cataract surgery. We did not exclude patients with BRVO because of poor VA of the affected eye. However, cases with Snellen VA of less than 20/40 in the fellow eye were excluded.

### Study examinations and treatments

At baseline, medical history was obtained from each patient. All patients underwent a comprehensive ophthalmologic examination, including measurement of best-corrected VA (BCVA) using the Landolt chart, indirect ophthalmoscopy, slit-lamp biomicroscopy with a non-contact lens, and OCT imaging (Spectralis HRA + OCT; Heidelberg Engineering, Heidelberg, Germany; RS-3000, Nidek, Gamagori, Japan; 3D OCT-1, Topcon, Tokyo, Japan). We created a thickness map of the entire retina using the volumetric OCT scanning of the macula. Within the central subfield of the Early Treatment Diabetic Retinopathy Study grid, FT was defined as the mean distance between the vitreoretinal interface and retinal pigment epithelium.

In addition to OCT angiography, we performed FA (Optos 200Tx Imaging System, Optos PLC, Dunfermline, UK) to assess the retinal circulatory status. However, FA was not performed in patients who showed allergic reactions to the dye and those who did not provide consent for the FA examination.

All patients were treated for ME with intravitreal anti-VEGF injections of ranibizumab (0.5 mg Lucentis; Novartis Pharma, Tokyo, Japan) or aflibercept (0.5 mg Eylea; Bayer Pharma, Tokyo, Japan). After the initial treatment, each eye was examined every month, and further injections were administered on an as-needed basis when ME or serous retinal detachment was evident at the fovea on the OCT images.

Follow-up examinations were performed at one (month 1) and three months (month 3) after the initiation of anti-VEGF treatment. At each follow-up, the BCVA was measured, and retinal morphology was examined using the OCT.

### Evaluation of metamorphopsia

At baseline, month 1, and month 3, we quantified metamorphopsia in the affected eyes and binocular metamorphopsia using the M-CHARTS^®^ (Inami, Tokyo, Japan). An M-CHARTS^®^ score of 0 indicates the absence of metamorphopsia. An M-CHARTS score of 0.3–0.5 or higher has been reported as the threshold for detecting metamorphopsia in daily life [23].

The M-CHARTS included 19 dotted lines. Each dot size was 0.1°, and the dot intervals ranged from 0.2 to 2.0° of the visual angle. A fixation point of 0.3° was placed at the center of each line. The examination was performed at a distance of 30 cm (with refraction correction) using charts with dotted lines (fine to coarse incremental spacing) that were shown to the patients one after another. When the patient recognized the presented line as straight, the visual angle to that line was considered as the degree of metamorphopsia. M-CHARTS were presented to the patient in vertical direction, followed by horizontal, vertical and horizontal scores were measured, and the average score of both directions was considered as the M-CHARTS score for the eye. In each patient, we evaluated metamorphopsia in the affected eye relative to the contralateral eye, and the binocular metamorphopsia was quantified using the M-CHARTS. Since the examinations were performed for the affected eye and the fellow eye, the sides of the affected eyes were disclosed by the examiners; however, the presence or absence of ME after the treatment and the treatment history were masked. The examiner was masked regarding the treatment history and recurrence of macular edema.

### Statistical analysis

The data are presented as the mean ± standard deviation, where applicable. The BCVA was converted to the mean logarithm of the minimum angle of resolution (logMAR) for statistical analyses. Time-point and group comparisons were performed using the paired and unpaired *t*-tests, respectively. Multivariate linear regression analysis was performed to evaluate the contribution of each initially identifiable factor to binocular metamorphopsia at baseline. Statistical analyses were performed using the SPSS statistical software (version 26.0.0, IBM Japan, Tokyo, Japan), and statistical significance was defined as *p* < 0.05.

## Results

### Baseline

At baseline, all eyes with unilateral BRVO showed the ME-associated visual disturbances. The logMAR VA and FT were 0.48 ± 0.38 (range in Snellen VA was 20/1000–20/13) and 550.4 ± 218.0 µm, respectively (Table 1). Of the 87 patients, 53 (60.9%; mean M-CHARTS score 0.29 ± 0.37) and 7 (8.0%; M-CHARTS score 0.02 ± 0.09) patients showed metamorphopsia in the affected eyes and binocular metamorphopsia, respectively.

**Table 1:** Characteristics of included patients with unilateral branch retinal vein occlusion

Baseline	
Number (patients)	87
Age (years)	68.4 ± 11.6
Sex (men/women)	41/46
Duration from onset of visual disturbance (months)	1.7 ± 4.3
Diseased eyes	
LogMAR visual acuity	0.48 ± 0.38
Snellen visual acuity, range	20/1000–20/13
Foveal thickness (µm)	550.4 ± 218.0
Height of foveal retinal detachment (µm)	95.4 ± 133.4
Defect length of foveal ellipsoid zone band (µm)	697.5 ± 685.5
Patients having metamorphopsia, n (%)	53 (60.9%)
M-CHARTS^®^ score	0.29 ± 0.37
Fellow eyes	
LogMAR visual acuity of fellow eye	− 0.03 ± 0.11
Snellen visual acuity of fellow eye, range	20/30–20/13
Patients having binocular metamorphopsia, n (%)	7 (8.0%)
M-CHARTS score for binocular vision	0.02 ± 0.09

Month 1		
Diseased eyes		
LogMAR visual acuity	0.27 ± 0.30	< 0.001
Snellen visual acuity, range	20/40–20/13	
M-CHARTS score	0.33 ± 0.37	0.322
Patients having metamorphopsia for diseased eyes, n (%)	59 (67.8%)	
Patients having binocular metamorphopsia, n (%)	11 (12.6%)	
M-CHARTS score for binocular vision	0.04 ± 0.13	0.164

Month 3		
Diseased eyes		
LogMAR visual acuity	0.19 ± 0.28	< 0.001
Snellen visual acuity, range	20/33–20/13	
M-CHARTS score	0.36 ± 0.44	0.140
Patients having metamorphopsia for diseased eyes, n (%)	55 (63.2%)	
Patients having binocular metamorphopsia, n (%)	9 (10.3%)	
M-CHARTS score for binocular vision	0.03 ± 0.10	0.622

Data are presented as the mean ± standard deviation unless otherwise indicated. Paired t-tests were used for the comparisons of baseline parameters with those at month 1 and month 3. LogMAR, logarithm of the minimum angle of resolution

### Changes in VA, FT, and metamorphopsia from baseline to month 3

**Fig. 1:**
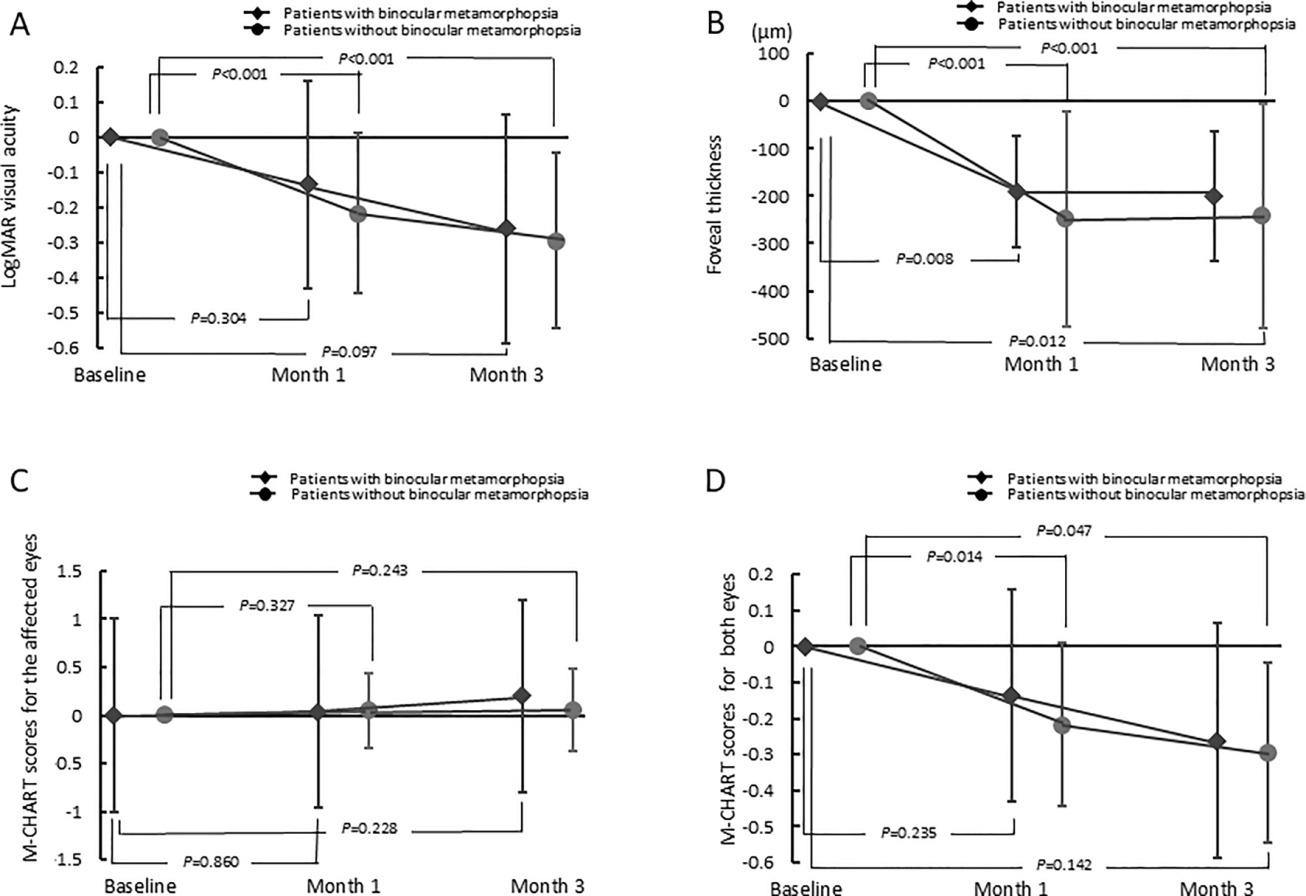
Changes in visual acuity, foveal thickness, and M-CHARTS^®^ scores for the affected and both eyes from baseline to month 3 after initiating anti-vascular endothelial growth factor treatments for branch retinal vein occlusion-associated macular edemaChanges in **a** visual acuity, **b** foveal thickness, **c** M-CHARTS score for the affected eyes, and **d** M-CHARTS score for both eyes

After the initiation of anti-VEGF treatment, the logMAR VA improved significantly relative to the baseline value (*p* < 0.001 at both months 1 and 3, relative to baseline) (Table 1, Fig. 1); however, the mean M-CHARTS scores in the affected eyes showed no improvements (0.33 ± 0.37 (*p* = 0.322) and 0.36 ± 0.44 (*p* = 0.140) at months 1 and 3, respectively) (Fig. 1). During the observation period, binocular metamorphopsia was resolved in 4 patients but developed in 6 other patients. At the end of the follow-ups (month 3), 9 (10.3%) patients had binocular metamorphopsia. The mean binocular metamorphopsia M-CHARTS scores were 0.04 ± 0.13 and 0.03 ± 0.10 at months 1 and 3, respectively, with no improvements relative to the baseline score (*p* = 0.164 and *p* = 0.622 for months 1 and 3, respectively) (Fig. 1).

### Factors associated with metamorphopsia for binocular vision

Table 2 shows the results of the multivariate linear regression analysis on the association of the binocular metamorphopsia and other factors at baseline. The M-CHARTS score representing binocular metamorphopsia was significantly associated with the metamorphopsia in the affected eye (95% confidence interval of 0.021–0.122 [*β* = 0.306, *p* = 0.006]).

**Table 2:** Multivariate analysis examining factors associated with metamorphopsia for binocular vision at baseline

	Metamorphopsia for binocular vision		
Baseline parameters	*β*	95% confidence interval	*P*-value
Age	0.068	− 0.002–0.003	0.619
Duration from onset of visual disturbance (months)	− 0.033	− 0.005–0.004	0.757
LogMAR visual acuity	− 0.070	− 0.086–0.054	0.652
Foveal thickness	− 0.078	0.000–0.000	0.598
M-CHARTS score	0.306	0.021–0.122	0.006
LogMAR visual acuity of fellow eyes	0.069	− 0.144–0.249	0.596

Online Resource 1 presents a comparison of clinical parameters between the patients with and without binocular metamorphopsia; the M-CHARTS score (0.64 ± 0.53 and 0.89 ± 0.53 at baseline and month 3, respectively) for patients with binocular metamorphopsia was significantly different (*p* < 0.001 at both months 1 and 3) from that for patients without defects (0.26 ± 0.34 and 0.28 ± 0.38 at baseline and month 3, respectively)

## Discussion

This study showed that in patients with BRVO-ME, a high degree of the (monocular) metamorphopsia in the affected eye may lead to binocular metamorphopsia. The retinal area affected by BRVO is commonly asymmetrical in the vertical direction. Retinal edema also includes or excludes the fovea depending on the vertically asymmetric affected-retinal area. In this study, we therefore performed the M-CHARTS test in the vertical and horizontal directions and used the average value as the patient’s score. The M-CHARTS gradually changes from narrowly spaced dotted lines to widely spaced dotted lines and looks for the point at which the patient no longer perceives metamorphopsia. The process is very simple, and there appears to be no learning curve in the implementation of M-CHARTS.

M-CHARTS scores have been used to quantitatively examine the metamorphopsia in various retinal diseases. Previous examinations using M-CHARTS showed that a significant percentage of patients with BRVO-ME complain of metamorphopsia in the affected eyes [14], which was reportedly associated with presence of cystoid spaces in the inner retina and/or whole retinal thickness at the fovea [14, 15]. However, a direct mechanism responsible for the emergence of metamorphopsia remains obscure mainly due to presence of various pathologies (other than the retinal exudative changes) at the macula of eyes. In eyes with BRVO, retinal ischemic changes and/or foveal photoreceptor damage might lead to (monocular) metamorphopsia [18, 24–26]. VA significantly improved after 3 months (*p* < 0.001, Table 1); however, the rate of the metamorphopsia of the affected eye significantly increased from baseline to month 3. In six patients who had no binocular metamorphopsia at baseline, binocular metamorphopsia developed at 3 months. The rate of binocular metamorphopsia significantly increased from baseline to 3 months (*p* = 0.022, data not shown). Metamorphopsia might occur when cellular alignments in the neuronal and glial cells become irregular, and the pathologic changes are persistent to some degree [14]. Recovery of the resolution of ME might be sufficient for the improvement of VA; however, it might be insufficient for the improvement of metamorphopsia. Insufficient recovery of metamorphopsia might involve persistent and irregular alignments of the neuroglial cells. In this study, the degree of metamorphopsia for the affected eye at month 3 was significantly and positively associated with the defect length of the foveal ellipsoid zone band at baseline (*p* = 0.022, data not shown); however, it was not associated with FT.

Previous investigations showed that, in human vision, binocular summation and inhibition occur when the monocular acuities of the two eyes are nearly equivalent and discrepant, respectively [27–29]. Hence, binocular summation and/or inhibition might have contributed differently to the binocular vision of patients included in this study because the VA improved significantly during the observation period. Although details of the visual compensation in this study were unknown, the rate of occurrence of the binocular metamorphopsia was approximately one-sixth to that of the metamorphopsia in the affected eyes, and the M-CHARTS score representing the binocular metamorphopsia was significantly lower than that of the monocular metamorphopsia in the affected eyes (*p* < 0.001, Online Resource 1.), suggesting that the deteriorated signal from the affected eye may be effectively processed for the binocular vision. Metamorphopsia for binocular vision could be detected using M-CHARTS; however, the degree of metamorphopsia might be so subtle that it was imperceptible in daily life.

This study had some limitations. First, the observation period was short, and hence, it was difficult to assess the long-term prognoses of the mono- and binocular metamorphopsia. Second, we did not evaluate the impact of the mono- and binocular metamorphopsia on the visual disabilities at the individual level that contribute to tasks such as reading and face discrimination.

In conclusion, using M-CHARTS, this study showed that a high degree of metamorphopsia in the affected eyes could cause binocular metamorphopsia. Further prospective studies are needed to understand the effects of binocular metamorphopsia on the long-term course, effects of treatments, and impact on visual disability.

## Supplementary Information

Below is the link to the electronic supplementary material.

Supplementary file1 (DOCX 24 KB)

## Data Availability

The datasets generated and/or analyzed during the current study are available from the corresponding author upon reasonable request.
