# Supplementary material for: Binocular metamorphopsia in patients with branch retinal vein occlusion: a multi-center study
Source: Int Ophthalmol. 2023 May 25;43(9):3279–86. doi: 10.1007/s10792-023-02731-0 (PMC10400688; doi:10.1007/s10792-023-02731-0)
Supplement: Supplementary file 1 — Supplementary file1 (DOCX 24 KB) [file 10792_2023_2731_MOESM1_ESM.docx]

***International Ophthalmology***

**Title: Binocular Metamorphopsia in Patients with Branch Retinal Vein Occlusion: A Multi-center Study**

**Authors:** Rie Osaka, Yuki Muraoka, Daisuke Nagasato, Yoshinori Mitamura, Naomi Nishigori, , Tomoaki Murakami, Kiyoshi Suzuma, Hitoshi Tabuchi, Akitaka Tsujikawa

***Corresponding Author:** Yuki Muraoka

Department of Ophthalmology, Kyoto University Graduate School of Medicine, Sakyo-ku, Kyoto 606-8507, Japan

E-mail: [muraoka@kuhp.kyoto-u.ac.jp](mailto:muraoka@kuhp.kyoto-u.ac.jp)

**Online Resource 1**. Comparisons of clinical parameters between patients with and without metamorphopsia for binocular vision

|  | Binocular metamorphopsia at baseline | | *P* value |
| --- | --- | --- | --- |
|  | Absence | Presence |  |
| **Baseline** | N=80 | N=7 |  |
| Age (years) | 68.1 ± 12.0 | 71.3 ± 6.7 | 0.491 |
| Sex (men/women) | 37/43 | 4/3 | 0.740 |
| Duration from onset of visual disturbance (days) | 49.7 ± 123.8 | 36.1 ± 39.0 | 0.773 |
| LogMAR VA of diseased eye | 0.49 ± 0.38 | 0.41 ± 0.38 | 0.628 |
| Snellen visual acuity, range | 20/1000–20/13 | 20/133–20/22 | n.a. |
| Foveal thickness (µm) | 555.9 ± 223.2 | 487.4 ± 144.2 | 0.429 |
| M-CHART score for diseased eyes | 0.26 ± 0.34 | 0.64 ± 0.53 | <0.001 |
| LogMAR VA of fellow eye | -0.03 ± 0.11 | 0.03 ± 0.11 | 0.155 |
| Snellen visual acuity, range | 20/33–20/20 | 20/33–20/17 | n.a. |
| M-CHART score for binocular vision | 0.00 ± 0.00 | 0.28 ± 0.16 | <0.001 |
| **Month 3** | N=78 | N=9 |  |
| Age (years) | 68.3 ± 11.9 | 69.1 ± 9.1 | 0.839 |
| LogMAR VA of diseased eye | 0.20 ± 0.29 | 0.16 ±0.16 | 0.690 |
| Snellen visual acuity, range | 20/250–20/17 | 20/200–20/13 | n.a. |
| Foveal thickness (µm) | 314.8 ± 97.8 | 280.2 ± 73.9 | 0.308 |
| M-CHART score for diseased eyes | 0.28 ± 0.38 | 0.89 ± 0.53 | <0.001 |
| LogMAR VA of fellow eye | -0.03 ± 0.11 | -0.06 ± 0.11 | 0.465 |
| Snellen visual acuity, range | 20/33–20/13 | 20/29–20/13 | n.a. |
| M-CHART score for binocular vision | 0.00 ± 0.00 | 0.28 ± 0.19 | <0.001 |
| logMAR, logarithm of the minimum angle of resolution; VA, visual acuity; n.a., not applicable *Unpaired t-test was used in the comparisons between patients with and without binocular metamorphopsia. | | | |
